# Impact of bending deformation on a capacitive power transfer system

**DOI:** 10.1038/s41598-025-29265-y

**Published:** 2025-11-23

**Authors:** Kiran Peirens, Ben Minnaert, Amélie Chevalier

**Affiliations:** Department of Electromechanics Cosys-Lab, University of Antwerp, 2000 Antwerp, Belgium

**Keywords:** Energy science and technology, Engineering

## Abstract

Wireless power transfer enables reliable energy delivery to fully embedded devices, eliminating the need for physical connectors while supporting device miniaturization. Flexible wireless power transfer systems extend this potential to applications requiring mechanical compliance, such as powering implantable medical devices in anatomically challenging locations with reduced foreign-body sensation. Capacitive power transfer is a near-field wireless power transfer method that is often cited as tolerant to bending deformations. However, this claim is based on limited evidence derived from studies on non-resonant systems or systems operating over larger distances, leaving the bending tolerance of resonant systems at small transfer distances largely unexplored. This work presents a systematic evaluation of bending on resonant capacitive power transfer at small distances, quantifying the impact of bending deformation on both the capacitive link and overall system performance. The results reveal that while concentric bending has negligible impact, outward receiver and inward transmitter bending significantly affect the system performance. AC-analysis measurements show that outward receiver bending shifts the optimal resonance frequency and reduces the power transfer efficiency by 54.5%. With inward transmitter bending, frequency splitting occurs which enhances the maximum power transfer efficiency with 8.5%. The findings redefine the bending robustness of capacitive power transfer systems and provide insight into their suitability for powering flexible applications.

## Introduction

Wireless power transfer (WPT) is an emerging technology, which allows devices to charge without the need of a physical connection. This elimination enables devices to be fully embedded without the additional need to incorporate a large battery. With WPT, the battery of the embedded devices can be reliably recharged, facilitating device miniaturization while ensuring safety, durability and robustness^1–3^. This makes the technology appealing for various applications including wireless sensors^4^, robotics^5^, electrical vehicles^6^ and biomedical applications^7^.

While the use of WPT is well studied for various biomedical applications, such as cardiac pacemakers^8^, drug pumps^9^, retinal stimulators^10^ and other neural stimulators^11^, its integration remains limited to only a few commercially-available systems, including cochlear implants (Synchrony 2 cochlear implant, MED-EL, Innsbruck Austria) and spinal cord simulators (Intellis neurostimulator, Medtronic, Minneapolis, USA). Both applications use inductive power transfer (IPT) which is the most established WPT technology, yet its conventional rigid form factor hinders broader adoption in biomedical settings, as it can cause foreign body sensation and discomfort for the patient^12^. To improve the bio-mechanical compatibility of IPT systems, the use and design of flexible IPT systems are explored, enabling implantation in more varied and anatomical challenging locations.

While rigid IPT systems are well-established in various applications, flexible IPT systems remain in the research and development phase due to unresolved challenges in reliability, durability, and manufacturing. With bending deformation, the mutual inductance of the IPT system changes, causing a shift in the system’s resonance frequency that degrades the power transfer capabilities^13–17^. Repetitive bending has further been shown to degrade performance, revealing a trade-off between achieving high efficiency and maintaining mechanical durability^18,19^. Several studies further highlight the ongoing fabrication challenges of flexible coils, where material limitations and process complexities hinder the manufacturing of flexible IPT systems^18–21^.

Capacitive power transfer (CPT) is an alternative WPT method that offers a promising alternative to wirelessly power applications prone to bending deformation. While IPT relies on the magnetic field, CPT uses the electric field, leading to a reduction in eddy current power losses, some tolerance to transmitter receiver-misalignment, and the ability to transfer energy through metal surfaces^22–24^. CPT systems are also reported to have a better tolerance against bending deformation and to offer a simpler fabrication process, which makes them especially interesting for flexible applications such as subcutaneous biomedical applications^25–29^. The claim regarding the tolerance against bending deformation is however ambiguous, as the effect of bending deformation on the capacitive link is only assessed twice in literature. Jegadeesan et al. ^25^ explored the influence of moderate concentric bending on a non-resonant CPT system for subcutaneous implants. Despite the reported drop in power transfer efficiency (PTE) and power dissipated in load (PDL) of approximately 25% and 60 mW, respectively, they described the effect of bending as minimal. In realistic applications, the imposed bending deformation is not restricted to the concentric case and can alter the average transfer distance, resulting in vertical misalignment that impacts system performance. Fang et al. ^27^ examined the effect of moderate symmetrical inward and outward plate bending on a resonant CPT system with a large transfer distance of 100 mm. CPT is, however, generally considered for applications with small transfer distances, where bending deformation will have a more pronounced effect on the average transfer distance^11,30,31^. Given the large transfer distance studied by Fang et al., their reported results are not directly applicable nor relevant to CPT systems that operate at small distances. Due to the absence of further research, it is impossible to estimate the potential of flexible CPT systems to wireless power applications that are susceptible to bending deformation such as subcutaneous biomedical implants, prosthesis, flexible wearable devices, robotic joints etc.

This work studies the impact of bending deformation on the capacitive link and power transfer performance of resonant CPT systems with short transfer distances. Through simulations and experimental validation, the effects of concentric, outward receiver, and inward transmitter bending on the capacitive link model are quantified for various transfer distances. In concentric bending, the average transfer distance remains unchanged, facilitating a translation of Jegadeesan et al.’s results to resonant CPT systems. For outward receiver and inward transmitter bending, the average transfer distance changes as one side deforms while the other remains planar. Although these deformations are less likely to occur, they define outer limits, with the actual deformation expected to have a smaller impact. To evaluate the power transfer performance under bending, the capacitive link is fabricated from two flexible printed circuit boards and integrated into a resonant CPT system. The results indicate that the impact of bending deformation can not be ignored, and should be carefully evaluated in the design of flexible resonant CPT systems with a small transfer distance. The main contributions of this paper are:

- Quantification of the impact of concentric, outward receiver, and inward transmitter bending on the equivalent pi-model capacitances of a CPT system with a small transfer distance.
- Analysis of how the variations in the capacitive link model affect the power transfer performance.
- Demonstration that while concentric bending has a negligible influence, the impact of inward transmitter and outward receiver bending are significant.

The remainder of this paper is organized as follows. The ”Methods” section describes the simulation setup and defines the bending deformations under study. The ”Influence on capacitive link” section presents finite element simulations that quantify the impact of bending deformation on the capacitive link, together with experimental measurements for validation. In the ”Influence on system performance” section, the capacitive link is integrated into a resonant CPT system, and its power transfer performance is evaluated under various bending conditions.

## Methods

To quantify the influence of plate bending deformation on the coupling behavior of a CPT system, the capacitive link is simulated in the electromagnetic finite element simulation software CST Studio Suite. Within the simulation environment, a four-plate CPT system is modeled as a two-port network, from which the admittance matrix and corresponding pi-model capacitances are extracted under different bending conditions. This simulation-based approach avoids the mathematical complexity of estimating the individual coupling capacitances of the capacitive link, which becomes impractical under bending deformation due to non-uniform electric field distributions and non-negligible parasitic effects. Standard capacitance equations rely on the assumption of parallel-plate geometries and uniform electric fields, assumptions that no longer hold once bending is introduced. Developing an analytical model would therefore require the use of more fundamental techniques, such as conformal mapping^32,33^. However, analytical formulations derived from such methods are highly system-specific^33,34^, remaining valid only for a single configuration and requiring re-derivation for any change in geometry or bending angle.

**Fig. 1:**
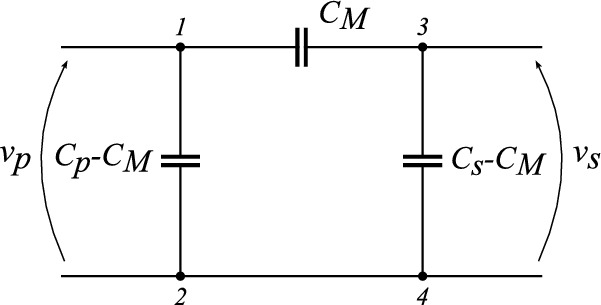
Electrical schematic of the equivalent pi-model.

In a four-plate CPT system, energy is transferred through a time-varying electric field established between two parallel-plate capacitances. Accurately characterizing this link is challenging because parasitic field interactions introduce unintended pathways that modify the effective coupling behavior of the system. In this work, the capacitive link is modeled by its equivalent pi-model (Fig. 1)^35^, consisting of a mutual coupling capacitance ($C_M$) to model the main coupling and two leakage capacitances ($C_P$ and $C_S$) connected to the transmitter and receiver, respectively.

**Fig. 2:**
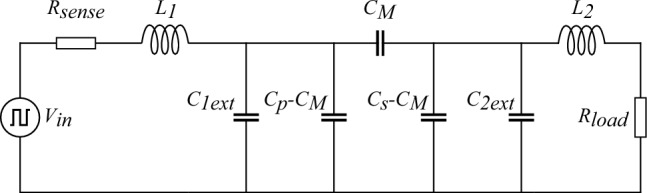
Electrical schematic of the resonant CPT system.

The capacitive link analyzed in this work consists of a two-plate transmitter and receiver, each with plate dimensions of 99 mm 99 mm and an inter-plate gap of 1 mm. Although the initial planar transfer distance *d* is altered in the simulation results, a transfer distance of 5 mm is used for the validation set-up. To evaluate the impact of bending deformation on the system’s power transfer performance, the studied capacitive link is integrated into a series-series resonance circuit, which is a well used compensation strategy to enhance the CPT performance in biomedical applications^26,28^. The resulting CPT system is shown in Fig. 2 and is powered by a half bridge inverter. It consists of a primary serie resistance ($R_{sense}$) to measure the input current, a primary and secondary compensation inductance ($L_1$, $L_2$), two external leakage capacitances ($C_{1ext}$, $C_{2ext}$), the capacitive link model ($C_M$, $C_P$ and $C_S$) and a load resistance ($R_{Load}$).

**Fig. 3:**
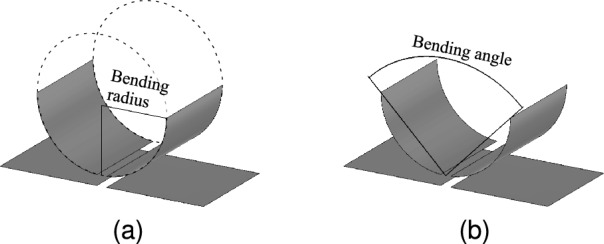
Definition of the bending deformation using (**a**) the bending radius and (**b**) the bending angle.

Precise assessment of bending deformation requires a clear definition. In prior literature, bending deformation is typically characterized using either the bending radius^14,25^ or the bending angle^16,36^. The first approach uses the bending radius, defined by the radius of a fictional cylinder around which the transmitter or receiver is wrapped (Fig. 3a). This method is not intuitive, as it heavily depends on the dimensions of the studied system. The second approach defines the degree of bending using a bending angle, measured from the center of the transmitter or receiver and between their edges (Fig. 3b). Unlike the bending radius, the planar reference configuration is not placed at infinity but is defined at a bending angle of 180°, which can be depicted graphically. The bending angle is also dimension-independent, allowing a straightforward comparison and translation of the obtained results to other systems subjected to cylindrical bending deformation.

On the capacitive link, an infinite range of bending deformations can be imposed. Given the impracticality of examining all possible bending deformations, specific boundary conditions concerning the bending position, type, and orientation are defined to ensure a manageable scope:

- The bending deformation is applied at the center of the transmitter and/or receiver.
- Only cylindrical bending is considered.
- The bending direction should align with either the X- or Y-axis.

**Fig. 4:**
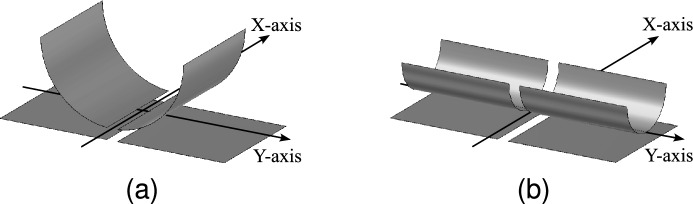
Definition of the bending along (**a**) the X-axis and (**b**) the Y-axis.

The X-axis bending direction is oriented along the inter-plate gap of the two-plate transmitter and receiver, while the second bending axis (Y-axis) is defined perpendicular to the X-axis and passes through the center of the transmitter or receiver plates. Figure 4 illustrates how receiver bending can be applied along the two axes while complying with the boundary constraints.

**Fig. 5:**
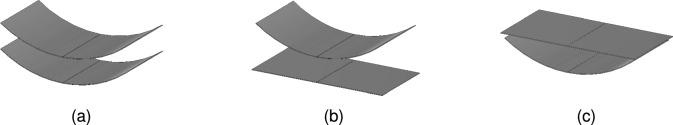
Definition of the considered bending deformations (**a**) concentric bending (**b**) outward receiver bending and (**c**) inward transmitter bending.

This work mainly focuses on three boundary bending deformations: concentric bending (Fig. 5a), outward bending of the receiver (Fig. 5b), and inward bending of the transmitter (Fig. 5c).

**Fig. 6:**
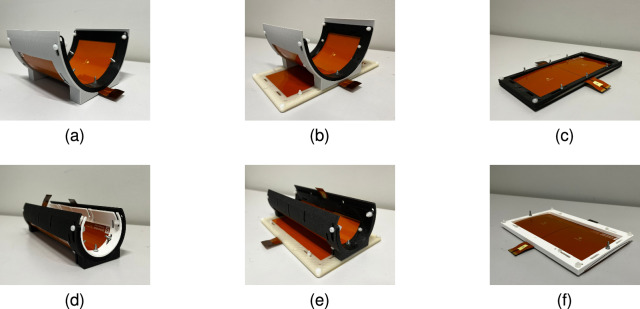
Maximum (**a**,**d**) concentric, (**b**,**e**) outward receiver, and (**c**,**f**) inward transmitter bending deformations considered under X- and Y-axis bending, respectively.

For concentric bending, the transfer distance remains constant. In outward receiver bending, the outer edges of the receiver are bent away from a planar transmitter, increasing the average transfer distance. The opposite occurs for inward transmitter bending, where the transmitter edges bent towards a planar receiver and the average transfer distance decreases. Bending situations where the transmitter and receiver are bent toward or away from each other are not considered in this work, as they are estimated to be rare or physically infeasible for the targeted applications. In this study, bending is primary considered as an undesirable but unavoidable phenomenon affecting the power transfer link. The focus therefore lies on applications such as biomedical implants, where a flexible power transfer link is desirable to enhance the bio-mechanical compatibility. While practical biomedical applications typically involve bending angles much smaller than 90° , the analysis is extended to a minimum bending angle of 90° (Fig. 6), as these extreme curvatures may occur in other application domains, such as: wireless power transfer systems for underwater environments^37^, unmanned aerial vehicles^38^, and consumer electronics^39^. Extending the analysis to this limit ensures that the results remain relevant to biomedical applications while also providing design insights for curved CPT systems in broader application areas, by establishing a predictive mapping between curved geometries and their equivalent planar configuration. For inward transmitter bending, the minimum bending angle is further constrained to prevent physical contact between the transmitter and the receiver. An overview of the maximum concentric, outward receiver and inward transmitter bending deformations is provided in Fig. 6.

## Results

### Influence on capacitive link

**Fig. 7:**
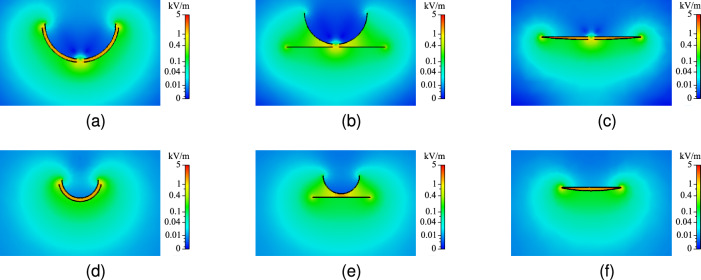
Simulated electrical field when the receiver is placed above the transmitter and at the maximum bending deformation for (**a**,**d**) concentric, (**b**,**e**) outward receiver bending, (**c**,**f**) inward transmitter X- and Y-axis bending, respectively.

With the defined boundary conditions, the impact of bending on the electric field is analyzed. While concentric bending preserves an uniform field, outward receiver and inward transmitter bending lead to non-uniform field distributions. An overview of the simulated electric fields for maximum concentric-, outward receiver-, and inward transmitter bending deformation is provided in Fig. 7.

**Fig. 8:**
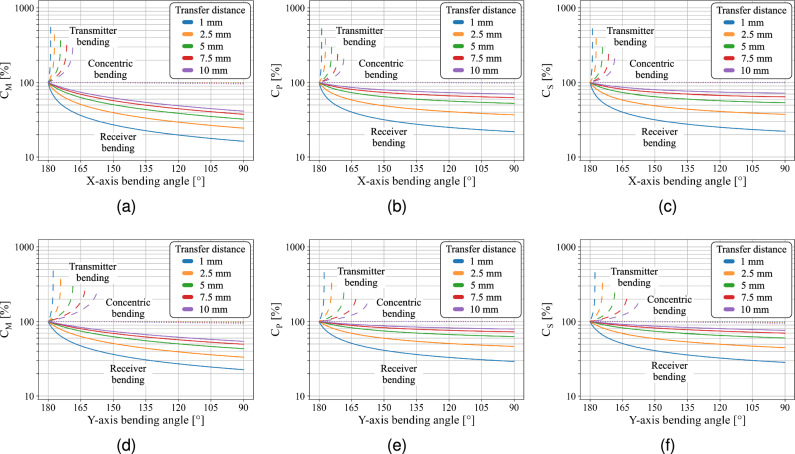
Quantification of the effect of concentric, outward receiver, and inward transmitter X- and Y-axis bending on the pi-model capacitances: (**a**,**d**) mutual capacitance $\mathrm {C_M}$, (**b**,**e**) primary capacitance $\mathrm {C_P}$, and (**c**,**f**) secondary capacitance $\mathrm {C_S}$. Concentric bending exhibits negligible influence, while outward receiver and inward transmitter bending have a significant impact.

Since the initial planar transfer distance *d* could influence the simulation results, the effect of bending deformation on the coupling behavior is simulated for transfer distances of 1, 2.5, 5, 7.5, and 10 mm. Figure 8 shows that concentric bending has a negligible impact on the pi-model capacitances independent of the initial planar transfer distance. For both inward transmitter and outward receiver bending, the impact of bending deformation is most pronounced in the mutual coupling capacitance ($C_M$) and reduces with increasing transfer distance. To make the results applicable beyond the capacitive link used in this work, all presented capacitance values are normalized to those of the matching planar reference configuration.

The results of Fig. 8 identify that plate bending significantly affects the pi-model capacitances of the capacitive link. Inward bending of the transmitter reduces the average transfer distance, which increases the mutual ($C_M$), primary ($C_P$), and secondary coupling capacitances ($C_S$). Conversely, outward receiver bending increases the transfer distance, reducing all pi-model capacitances. In concentric bending, the average transfer distance remains constant, which renders its impact on the capacitive link negligible. Around the bending angle of 90°, a slight decrease in coupling capacitance occurs, which is caused by a small reduction in plate overlap. Considering the influence of bending direction, X-axis bending causes larger variations in all pi-model capacitances compared to Y-axis bending. At larger plate distances, the influence of bending diminishes as the relative change in transfer distance becomes smaller.

### Verification measurements

The simulation results are validated using a physical CPT system that matches the dimensions of the simulated link. The capacitive link is created from two flexible printed circuit boards which serve as the two plate transmitter and receiver. To mimic the bending deformation, the flexible capacitive link is mounted on 3D printed shapes. For the verification measurements a reference planar transfer distance *d* of 5 mm is maintained. The individual coupling capacitances are measured according to the methodology defined by Huang et al.^35^. To measure the primary and secondary coupling capacitance, the secondary and primary side are short circuited sequentially, while the input capacitance is measured with a Hioki IM3536 LCR-meter. For the mutual coupling capacitance, the primary side is connected to a Rigol DG882 PRO signal generator. While applying a sinusoidal voltage, the voltage is measured at the primary and secondary side using a TAO44 and TAO57 differential probe, respectively. With the measurement data, the mutual coupling capacitance is calculated in accordance with equation (1), where ${V_{so}}$ represents the peek to peek secondary output voltage and $V_{pp}$ the peek to peek primary input voltage.

1 $$\begin{aligned} C_{M} = \dfrac{V_{so}}{V_{pp}}(C_{P}+C_{probe}) \end{aligned}$$

**Fig. 9:**
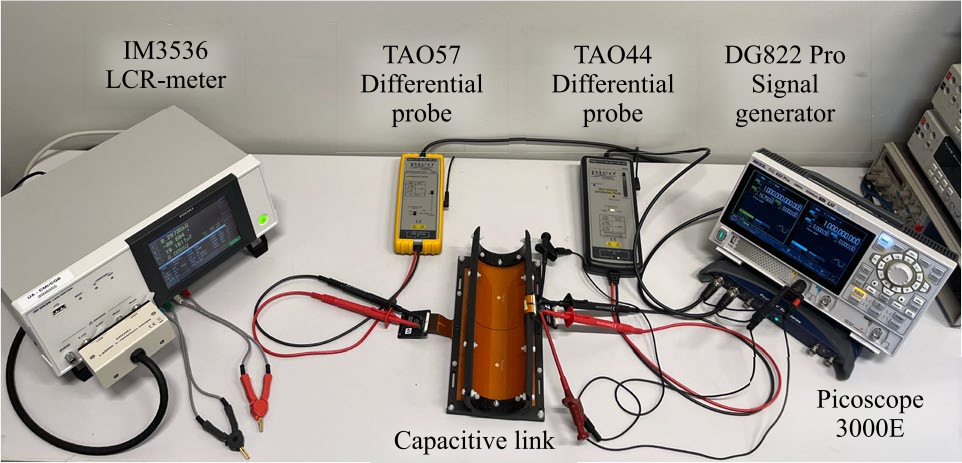
Measurement setup used to validate the simulated influence of bending on the pi-model capacitances.

**Fig. 10:**
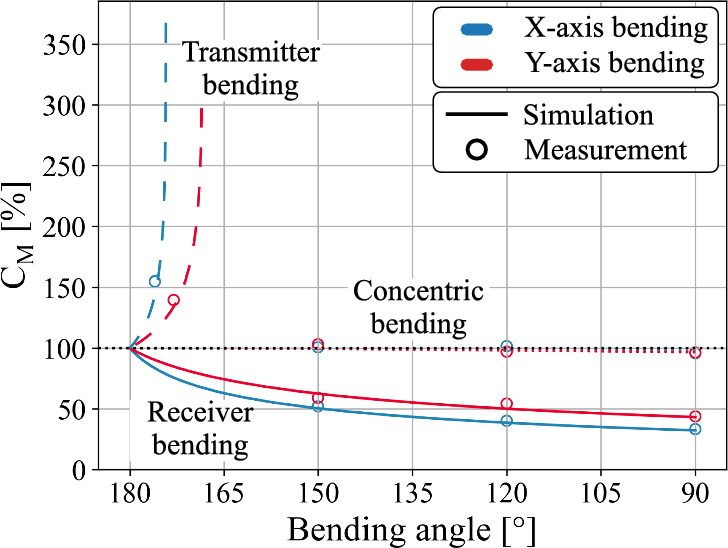
Validation of the simulated influence of bending deformation on the mutual coupling capacitance $\mathrm{C_M}$ for a CPT system with a planar transfer distance of 5 mm.

Figure 9 illustrates the measurement set-up used to determine the mutual coupling capacitance of the physical capacitive link under 90° of Y-axis outward receiver bending. With the proposed measurement set-up, the pi-model capacitances of the capacitive link are quantified under various bending conditions. Measurements are conducted for both X- and Y-axis outward receiver bending at bending angles of 150°, 120°, and 90°. The same angles are applied to characterize the capacitive link under concentric bending deformation. For X- and Y-axis inward transmitter bending, measurements are performed at a maximum bending angle of 176° and 140°, respectively. These angles ensure that a 1 mm gap remains between the edges of the transmitter and receiver plates. To directly compare the measurements with the simulation results, the measurements are also normalized using the corresponding capacitances measured in the planar configuration. For all measurements, a close agreement with the simulation results is observed, as visualized in Fig. 10.

The results indicate that the impact of bending deformation on the capacitive link of a CPT system cannot be considered negligible. Outward receiver bending refers to a deformation in which the edges of the two-plate receiver are bent away from the planar transmitter, thereby increasing the average transfer distance. In contrast, inward transmitter bending occurs when the outer edges of the two-plate transmitter are bent towards the receiver, resulting in a reduction of the average transfer distance. Under outward receiver bending, the pi-model capacitances exhibit a sharp initial decline, followed by a more gradual reduction at larger bending angles. This behavior is validated by the measured mutual coupling capacitance, which decreases by 48% and 41% at a bending angle of 150°, and by 67% and 56% at 90° for X- and Y-axis bending, respectively. Inward transmitter bending causes an opposite trend and increases the mutual coupling capacitance to 164% for 176° of X-axis bending and 136% for 140° of Y-axis bending. Despite the obtained increase in coupling capacitances, inward transmitter bending requires careful assessment as the local decrease in transfer distance creates areas with intensified electric field (Fig. 7). While the provided analysis enables the quantification of bending-induced variations in the coupling capacitances, the extent to which these variations affect the power transfer capabilities such as PTE and PDL are determined in the next section.

### Influence on system performance

The previous section indicated that bending deformation alters the capacitive link model of a CPT system. Since these capacitances directly define the power transfer characteristics, corresponding variations in PTE and PDL are to be expected. To quantify these effects, the studied capacitive link is integrated into a functional CPT system and evaluated under varying bending conditions.

The CPT system is built around the planar configuration, with a resonance frequency of 1.010 MHz. For the compensation strategy, a series-series compensation network is selected (Fig. 2), as this is commonly used in flexible biomedical applications. To reduce the size of the compensation inductors, external leakage capacitances of 150 pF are added to the primary and secondary side of the capacitive link, lowering the capacitive coupling factor to 0.05 for the planar configuration. The CPT system is powered by a zero-voltage-switching (ZVS) half-bridge Class D inverter. To maximize the PDL, a 46.7 $\Omega$ load resistor is connected at the secondary side to dissipate the AC output power. An overview of the schematic parameters with their corresponding values is provided in Table 1.

**Table 1:** Measured CPT system values.

Parameter	Description	Value
${V_{in}}$	Square input voltage	$18\,V$
${R_{sense}}$	Sense resistance	$9.97\,\Omega$
$L_{1}$	Primary inductance	$148\, \mu H$
$C_{1ext}$	Primary external capacitance	$151\, pF$
$C_{P}$	Primary coupling capacitance	$8.36\, pF \sim 17.3\, pF$
$C_{M}$	Mutual coupling capacitance	$2.42\, pF \sim 11.2\, pF$
$C_{S}$	Secondary coupling capacitance	$7.45\, pF \sim 16.9\, pF$
$C_{2ext}$	Secondary external capacitance	$143\, pF$
$L_{2}$	Secondary inductance	$155\, \mu H$
${R_{load}}$	Load resistance	$46.7\,\Omega$

**Fig. 11:**
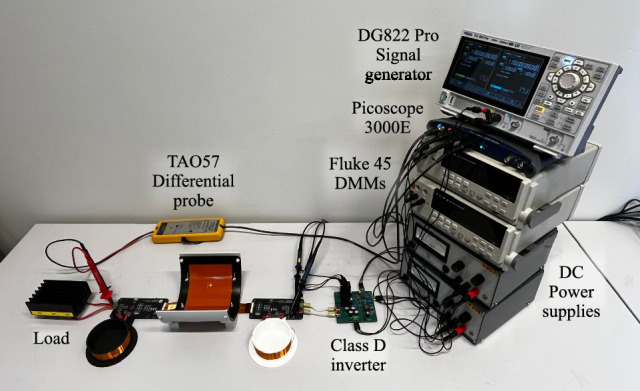
Measurement setup used to examine the impact of bending deformation on the power transfer performance.

Within the CPT system, the power is measured at multiple locations to accurately characterize the systems power flow (Fig. 11). The DC input power is captured using two FLUKE 45 digital multimeters (DMMs), measuring the input voltage and current respectively. To measure the AC input power a 9.97 $\Omega$ resistor is placed between the secondary side of the inverter and the capacitive link. Although this resistor reduces the efficiency and output power, it ensures accurate characterization of the AC input power even when bending-induced mismatches occur. The output power is measured across the load with a TAO57 differential probe. Figure 11 shows the designed CPT system integrated in the described power measurement set-up to monitor the power flow for 90° of concentric X-axis bending deformation.

With the measurement set-up, the DC-to-AC power flow of the reference planar configuration is characterized at an inverter output voltage of 18 V and an operating frequency of 1.010 MHz. Under these conditions, an output power of 2.77 W is achieved, with an inverter and AC-to-AC efficiencies of 83.3% and 74.4%, respectively.

To ensure generality and isolate the effects of bending deformation on the capacitive link, the remainder of this work focuses on the AC-to-AC behavior of the CPT system. Unlike DC-to-DC performance, which is strongly influenced by the choice of inverter and rectifier topologies, the AC-to-AC analysis directly reflects the intrinsic characteristics of the capacitive link. This approach enables a clearer understanding of how bending alters the capacitive coupling and power transfer capability, independent of the used inverter and rectifier.

**Fig. 12:**
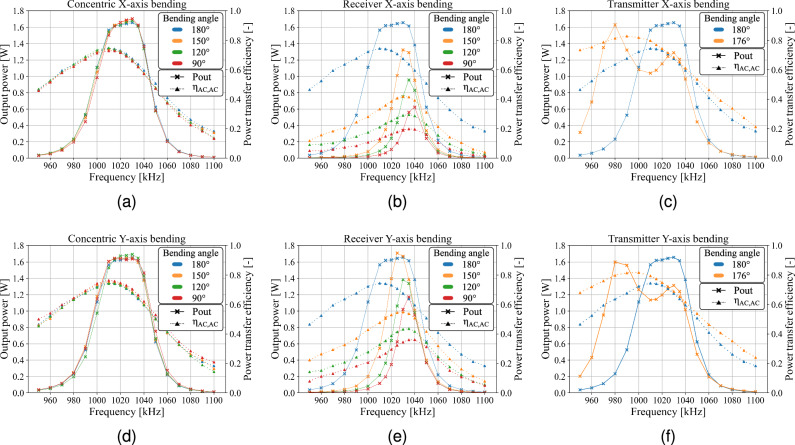
Variation in the output power (solid) and power transfer efficiency (dotted) under (**a**,**d**) concentric, (**b**,**e**) outward receiver, and (**c**,**f**) inward transmitter X- and Y-axis bending. Concentric bending has no effect, outward receiver bending reduces the power transfer performance, and inward transmitter bending improves it.

While the capacitive link is subjected to bending deformation, the AC-to-AC efficiency and output power are measured over a frequency range of 950 kHz to 1100 kHz. For consistency, the bending configurations previously used to analyze the capacitive link model are reconsidered, enabling a direct assessment of how the measured changes in pi-model capacitances impact the output performance. For both concentric and outward receiver bending, measurements are conducted at bending angles of 150° , 120° , and 90° along the X- and Y-axes. In the case of inward transmitter bending, angles of 176° and 140° are selected for X- and Y-axis deformation, respectively. The AC-analysis in Fig. 12 shows the measured efficiency and output power for each deformed configuration, and reveals that while concentric bending has a negligible impact, receiver and transmitter deformations lead to deviations from the 180° planar configuration.

Concentric bending along both the X- and Y-axes (Fig. 12a,d) has no significant impact on the systems output power, efficiency and optimal operating frequency. In both the planar and concentric bend configurations an output power of 1.6 W is maintained between 1010 and 1035 kHz and a peak efficiency of 74.4% at 1010 kHz is measured. Outward receiver bending (Fig. 12b,e) significantly reduces the power transfer capability and alters the frequency at which the peak efficiency occurs. At 90° of outward receiver bending, the maximum AC-to-AC efficiency drops to 19.9% at 1040 kHz for X-axis deformation and 36.1% at 1035 kHz for Y-axis deformation. With inward transmitter bending (Fig. 12c,f) frequency splitting is observed, leading to peak efficiency of 82.9% and 81.7% for X- and Y-axis deformation, respectively.

## Discussion

This work assesses the impact of bending deformation on the capacitive link model and the power transfer capabilities of CPT systems with small transfer distances. The results demonstrate that, while the influence of concentric bending is insignificant, the effects of inward transmitter and outward receiver bending are more pronounced and cannot be neglected.

For outward receiver bending the equivalent pi-model capacitances of the capacitive link model increases, while an opposite trend is observed for inward transmitter bending. These variations result from structural curvature, which introduces a non-uniform electric field, and changes the system’s average transfer distance. The non-uniform electrical field complicates the analytically quantification of the resulting capacitive link model, making measurements or a finite element analysis a more suitable approach for assessing the variations. The magnitude of the observed changes depends on the initial planar transfer distance, with shorter distances exhibiting greater sensitivity to bending deformation.

To evaluate how the variations in pi-model capacitances affect the power transfer capabilities, the studied capacitive link is integrated into an example CPT system. The analysis confirms that under concentric bending, the planar output performance is maintained, indicating that the findings of Jegadeesan et al.^25^ also hold for resonant systems. For both inward transmitter and outward receiver bending, the measured changes in the capacitive link model translate to observable variations in PDL and PTE (Fig. 12). Bending also affects the system’s resonant frequency, indicating that assessing the influence of bending on the primary and secondary coupling capacitances remains important despite the presence of external leakage capacitances. In literature, various compensation strategies have been proposed to counteract such frequency shifts, including high-order or adaptive impedance-matching networks^24,40^ and active frequency-control techniques^41^. Although these strategies can improve the CPT performance under bending deformation, a reduction in PTE and PDL will persist due to the presence of the external leakage capacitances which minimize the size of the required compensation inductance. These capacitances mitigate the effect of bending on the primary and secondary coupling capacitances, thereby increasing the bending sensitivity of the capacitive coupling factor, which defines the theoretical maximum PTE of the system^42^. The variation in coupling factor also affects the zero-voltage switching condition of the inverter, necessitating the implementation of an appropriate inverter control strategy to maintain minimal switching power losses^43^. To increase the output power, the input power could be increased. However, similar to inward transmitter bending, this would require careful evaluation, as it intensifies the electric field and may lead to non-compliance with safety regulations.

In literature, only few studies assess the influence of bending deformation on CPT systems. A direct comparison of the results is not possible as the obsereved influence depends on system parameters such as planar transfer distance, studied type of bending and plate dimensions. This is demonstrated in the work of Fang et al.^27^ where the impact of bending deformation on a CPT system with a large transfer distance is assessed as negligible. Their findings are confirmed in this work through simulations with varying transfer distances, which prove that the influence of bending deformation diminishes with increasing distance.

In the context of flexible CPT applications, such as biomedical implants, bending is considered as an undesired yet unavoidable phenomenon and is typically limited to only small deformations. Jegadeesan et al.^25^, for instance, reported a worst-case bending radius of 20 mm and noted that this deformation is already unlikely to occur in post implantation tissue environment. Considering the system dimensions used in their study, the specified bending deformation corresponds to an equivalent bending angle of 151° in our work. Despite the small estimated deformations, the impact on the CPT system performance should not be disregarded. As shown in Fig. 8, even minor bending deformations can significantly affect the different pi-model capacitances, as they exhibit a sharp initial decline under single outward receiver bending. This trend is reflected in Fig. 12, where a PTE reduction of 32.6% and a PDL reduction of 330 mW are observed under 150° of single outward receiver bending along the X-axis, while assuming a planar reference separation distance of 5 mm and active frequency tracking to compensate for the resonance frequency shift. The results in Fig. 8 further indicate that the measured performance degradation intensifies for smaller reference planar transfer distances, as bending deformation exerts a stronger influence on the pi-model capacitances when the reference planar transfer distance is reduced. Although the results presented in Fig. 12 suggest that inward transmitter bending does not degrade the system performance, such configurations warrant careful consideration, as the associated local enhancement of the electric field (Fig. 7) may lead to non-compliance with established safety standards^44,45^.

The results exceed the biomedical application domain due to the considered range of bending deformations and therefor can be used for other applications^37–39^. Figures 8 and 12 allow predictive mapping between curved and planar configurations, enabling a reliable estimation of the system performance based on their equivalent planar model. The findings further demonstrate that concentric bent CPT structures can be designed using the equivalent planar configuration as no significant variation in pi-model capacitances or performance is observed. With Fig. 8, a similar predictive mapping approach can be used to estimate the variation in coupling capacitance under single inward transmitter or outward receiver bending. Rather than relying on complex analytical methods such as conformal mapping, the presented results provide a practical first-order assessment to estimate the coupling capacitances and overall performance of bent CPT systems.

The results of this work indicate that the impact of bending deformation can not be directly regarded as negligible, but greatly depends on the type of induced bending deformation an system dimensions. While inward transmitter bending increases the output performance, it also introduce regions of enhanced electrical field, which should be monitored to ensure compliance with bio-safety regulations^44,45^. To mitigate the negative effects of outward receiver bending, frequency splitting could be used in certain applications^27^. However, for size constrained applications, such as biomedical applications, frequency splitting can prove to be difficult, necessitating active control strategies. In realistic applications, bending deformations are likely to fall between concentric and inward transmitter or outward receiver bending. For these deformations the presented results should be interpreted as outer limits, with the actual impact of bending expected to be less pronounced.

## Conclusions

This work quantifies the impact of bending deformation on the performance of CPT systems with small transfer distances. Using simulations and experimental validation measurements, the influence of bending deformation on the capacitive link is quantified. The results show that while concentric bending has a negligible effect, outward receiver and inward transmitter bending significantly alter the capacitive coupling, with an impact more pronounced for smaller planar transfer distances. At system level, AC-analysis measurements revealed that outward receiver bending causes a shift in the optimal resonance frequency and a maximum PTE drop of 54.5%. Given the variations in PTE, PDL and resonance frequency, the incorporation of adequate control strategies is required to reduce the CPT system’s sensitivity to single outward receiver bending. Inward transmitter bending induced frequency splitting and enhanced the output performance, but also creates enlarged electric field regions that require careful management. While prior literature describes CPT as robust and insensitive to bending deformation, the presented results demonstrate that this robustness is not inherent but strongly depends on the type of bending deformation imposed. Although a direct comparison with other WPT methods is not possible, CPT remains the most effective WPT method to power flexible applications susceptible to concentric bending deformations, but loses effectiveness as the average transfer distance increases.

## Data Availability

The datasets generated and/or analyzed during the current research are available in the Zenodo repository, at https://doi.org/10.5281/zenodo.16995695.
